# Association of childhood acute lymphoblastic leukaemia with cancers in family members

**DOI:** 10.1038/sj.bjc.6602867

**Published:** 2005-11-15

**Authors:** E Couto, B Chen, K Hemminki

**Affiliations:** 1 Department of Biosciences at Novum, Karolinska Institute, 141 57 Huddinge, Sweden; 2 Division of molecular Genetic Epidemiology, German Cancer Research Center (DKFZ), Heidelberg, Germany

**Keywords:** childhood acute lymphoblastic leukaemia, family history, prenatal origin, testicular cancer

## Abstract

Children whose twins have had leukaemia have a higher risk of contracting acute lymphoblastic leukaemia (ALL), confirming a prenatal origin of the disease. This association was not true when considering other types of affected first-degree relatives. Children whose fathers were diagnosed with testicular cancer have a higher risk of ALL.

Although acute lymphoblastic leukaemia (ALL) is the most common neoplasia among children worldwide (Parkin *et al*, 1999), many aspects of its aetiology remain unclear. Ionising radiation, Down's syndrome and ataxia-telangietasia are established risk factor for childhood ALL (Greaves, 1997; Little, 1999). Possible associations between childhood ALL risk and infection, and prenatal factors have been suggested (MacMahon, 1992; Kinlen *et al*, 1990; Kinlen, 1995; Wiemels *et al*, 1999; Greaves *et al*, 2003; Stiller, 2004; Gilham *et al*, 2005). Results from epidemiological studies investigating the familial risk of childhood ALL are difficult to interpret, generally because of small sample size. Published studies have considered a wide range of cancers diagnosed in relatives, ranging from family histories of leukaemia (Buckley *et al*, 1996; Hemminki *et al*, 2001a); or more broadly, family histories of haematologic neoplasms (Perrillat *et al*, 2001; Infante-Rivard and Guiguet, 2004) and solid tumours (Perrillat *et al*, 2001). Results from these studies are inconclusive. Children with parents diagnosed with leukaemia were reported to have a similar risk of ALL as the general population (Hemminki and Mutanen, 2001b). A higher risk of leukaemia was observed among children with affected siblings (Hemminki *et al*, 2001a).

This population-based cohort study aims to investigate the risk of childhood ALL associated with a family history of leukaemia and other neoplasms in parents and/or siblings.

## MATERIAL AND METHODS

### Study population

Data from the Swedish Family-Cancer Database were used. This Database was described in detail previously (Hemminki *et al*, 2001c). Briefly, the Swedish Family-Cancer Database was created linking four different national sources of information: the multigeneration register, censuses, cancer registries and national deaths notifications. The multigeneration register includes people born in Sweden after 1931, and parents born in 1864 and after. Information from censuses of years 1960, 1970, 1980 and 1990 was also used. Cancers were identified trough the Swedish Cancer Registry that covers cancers diagnosed between 1958 and 2002. Information obtained through these sources was linked together using the national 10-digit personal number; this information was available for all the participants. Familial relationships were determined using the multigeneration register. 406 091 participants whose either parent's personal number was missing were excluded as sibships could not be determined. Only histories of cancers among mothers, fathers and siblings were considered.

Participants born between 1932 and 2002 were included, and cases of childhood ALL, (International Classification of Diseases, 7th revision, coding 204.0), diagnosed between 1961 and 2002, and aged less than 20 years old were considered.

### Statistical methods

Standardised incidence ratios (SIRs) for childhood ALL were calculated dividing the observed number of ALL by the expected number of cases. The SIRs were stratified by follow-up period (1961–1969, 1970–1979, 1980–1989, 1990–2002), and age using 5 years categories. 95% Confidence intervals (CI) were obtained assuming a Poisson distribution (Altman *et al*, 2000).

SIRs of childhood ALL were estimated comparing people with first-degree relatives diagnosed with leukaemia to the general population. This was also carried out considering 34 other type of cancers among relatives. Only results for cancer sites with five or more cases in children with affected relatives are reported here (18 out of 34).

## RESULTS

In the total population of 6 994 345 individuals, 1925 cases of childhood ALL were ascertained. Only 19 of those had a first-degree relative diagnosed with leukaemia, with a SIR for childhood ALL of 2.21 (95% CI: 1.33–3.45) (Table 1). However, this increase in risk remained statistically significant only when the affected relative was a twin. The SIR for childhood ALL in participants with twins diagnosed with any type of leukaemia was equal to 208.28 (54.05–641.09), compared to 1.27 (0.55–2.50) and 2.22 (0.51–7.32) when the affected relatives were parents and siblings (excluding twins), respectively. All affected twins were females. The SIR for childhood ALL was 2.18 (0.94–4.30) when first-degree relatives (excluding twins) were diagnosed with acute lymphoblastic leukaemia; when including twins, this SIR was equal to 3.80 (2.08–6.38). The SIR for childhood ALL when relatives were diagnosed with myeloid leukaemia was 1.25 (0.34–3.20) (Table 1).

When investigating the risk of childhood ALL associated with histories of other types of cancer among first-degree relatives, only children with parents diagnosed with testicular cancer showed an increase in risk. The SIR of childhood ALL for such offspring was 3.12 (95% CI: 1.50–5.75) (Table 2). Only the SIR for childhood ALL when parents were diagnosed with malignant testicular teratoma was statistically significant, 4.10 (1.29–9.64) compared to 2.26 (0.59–5.85) for seminoma. The five paternal teratomas were diagnosed between years 1982 and 1996 at ages 26–38 years: three of the fathers were diagnosed before conception of the child who was to be diagnosed with ALL.

## DISCUSSION

Children with twins diagnosed with leukaemia had a much higher risk of contracting acute lymphoblastic leukaemia than the general population. This increase in risk was not observed with other type of affected first-degree relatives, such as parents or siblings (excluding twins).

In this large study, the data were obtained through Swedish national registries. These registries were reported to be close to 100% complete (Centre for Epidemiology, 2000), including most of the Swedish population in the periods covered by these registries. The results reported in this article are, therefore, generalisable to the whole population. Familial relationships were determined using information from the multigeneration registry, which is more reliable than using self-reported family histories.

Previous publications using information from the same Database reported no increase in childhood ALL when parents were diagnosed with leukaemia (Hemminki and Mutanen, 2001b). A higher risk of leukaemia in people with siblings diagnosed with the disease was reported, but this investigation did not examine the risk associated with having affected twins (Hemminki *et al*, 2001a). The methods used in this article differ from previous publications using the same data. Indeed, four new more years of follow-up were added. Furthermore, a stricter criterion of selection for parents was carried out. In the present report, people with missing information for any parents were excluded, while previously offspring with information on at least one of the parents were considered. The present publication investigates further the association between having a father diagnosed with testicular cancer and the risk of childhood ALL by considering different histological types of testicular cancers.

This study adds substantially to previous studies, as it investigates the familial cancer risk of childhood ALL considering different types of leukaemia in affected first-degree relatives. Indeed, epidemiological studies have mainly considered family histories of other malignancies than ALL in first-degree relatives, such as solid tumours (Perrillat *et al*, 2001) and haematologic neoplasms (Perrillat *et al*, 2001; Infante-Rivard and Guiguet, 2004). In those studies, no statistically significant increase in risk of childhood ALL was found. Winther *et al* (2001) reported no increase in risk of leukaemia in people whose siblings were diagnosed with childhood leukaemia.

In this article, a very high risk of childhood ALL was found in people with twins diagnosed with leukaemia. All the twin pairs for whom at least one of them was affected were females, suggesting that they were monozygotic twins. Commonly, increases in cancer risk seen in people with close relatives diagnosed with cancer are suggested to be associated with shared genes and/or post-natal environment. However, it has been mentioned that due to the young age at onset of childhood ALL and the latency expected for cancer evolution, it is possible that the disease originates before birth (Greaves, 1999). It has been hypothesised that the sharing of placenta of monozygotic twins might contribute toward concordance of leukaemia (Greaves *et al*, 2003).

In the present study, when considering 18 different cancer sites diagnosed in first-degree relatives, an increase of childhood ALL was found only in participants with parents diagnosed with testicular cancer. Due to the large number of cancers considered, it is possible that the higher risk observed in these children is due to chance. An unproven explanation could be that the treatment of fathers for testicular cancer with cisplatin caused permanent damage to sperm which could contribute to the higher risk of ALL observed in offspring (Greene, 1992). When considering seminoma and teratoma histological types separately, an increase in risk of childhood ALL was observed among children whose fathers were diagnosed with malignant teratoma. This was not true for malignant seminoma. Among the five malignant teratomas diagnosed in fathers of children with ALL, three were diagnosed before conception of the child. Those testicular cancers were diagnosed in 1991 and 1996. The use of cisplatin to treat testicular cancer has started in the 1970s (IARC Monographs, 1981), and is still in use, hence the three malignant teratomas occurring before the birth of the child to be diagnosed with ALL were most probably all treated with cisplatin.

More studies are needed to investigate whether the increased risk of ALL observed in children with twins diagnosed with leukaemia is associated with common familial factors such as shared genes and/or postnatal environment, or if it is more explained by prenatal factors.

This population-based cohort study indicates that children with first-degree relatives diagnosed with leukaemia have a higher risk of contracting ALL. However, this increase in risk seems to exist only when the affected relative is a twin, confirming a prenatal origin of childhood ALL.

## Figures and Tables

**Table 1:** Standardised incidence ratios (SIRs) for childhood acute lymphoblastic leukaemia associated with a family history of leukaemia

**Family history of**	**Obs.^a^**	**SIRs^b^ (95% CI)**
*Any type of leukaemia (ICD* c *: 204–209), in:*		
Any first-degree relatives	19	2.21 (1.33–3.45)
		
Parents only	8	1.27 (0.55–2.50)
		
Siblings only	11	4.82 (1.70–12.20)
Siblings excluding twins	5	2.22 (0.51–7.32)
Twins	6	208.28 (54.05–641.09)
		
*Acute lymphoblastic leukaemia (ICD 204.0)*, *in*		
Any first degree relatives	14	3.80 (2.08–6.38)
Any first-degree relatives excluding twins	8	2.18 (0.94–4.30)
		
*Myeloid leukaemia (ICD 205), in*		
Any first degree relatives	4	1.25 (0.34–3.20)
Any first-degree relatives excluding twins	4	1.25 (0.34–3.20)

a Observed number of cases.

b SIRs stratified by age and follow-up period.

c International Classification of Disease.

**Table 2:** Standardised incidence ratios (SIRs^a^) for childhood acute lymphoblastic leukaemia in children with relatives diagnosed with different type of cancers

	**Relatives affected**					
	**Parents only**			**Siblings only**		
**Cancer in relatives**	**Obs.**	**SIRs**	**(95% CI)**	**Obs.**	**SIRs**	**CI (95% CI)**
Stomach	7	1.32	(0.53–2.73)			
Colon	11	0.89	(0.44–1.60)			
Rectum	5	0.67	(0.22–1.55)			
Pancreas	6	1.42	(0.52–3.08)			
Lung	18	1.26	(0.74–1.98)			
Breast	40	1.00	(0.71–1.36)	3	1.49	(0.22–6.17)
Cervix	7	1.02	(0.41–2.10)			
Endometrium	7	1.43	(0.57–2.94)	1	6.51	(0.12–51.34)
Ovary	9	1.51	(0.69–2.86)			
Prostate	24	1.09	(0.70–1.62)			
						
Testicular	10	3.12	(1.50–5.75)	1	0.87	(0.02–6.85)
Seminoma	4	2.26	(0.59–5.85)			
Teratoma	5	4.10	(1.29–9.64)			
						
Urinary bladder	9	1.00	(0.46–1.91)			
Melanoma	11	0.77	(0.39–1.39)	1	0.61	(0.01–4.80)
Nervous system	11	0.95	(0.47–1.69)	3	0.92	(0.13–3.78)
Thyroid gland	4	1.06	(0.29–2.72)	1	1.95	(0.03–15.39)
Connective tissue	5	2.38	(0.77–5.56)	1	2.08	(0.04–16.37)
Non-Hodgkin's disease	7	0.88	(0.35–1.82)			
Hodgkin's disease	4	1.82	(0.50–4.65)	2	2.31	(0.20–11.83)

a SIRs stratified by age and follow-up period.
